# Relationship between physical activity time and intramuscular adipose tissue content of the thigh muscle groups of younger and older men

**DOI:** 10.1038/s41598-021-99126-x

**Published:** 2021-10-05

**Authors:** Madoka Ogawa, Noriko Tanaka, Akito Yoshiko, Yoshiharu Oshida, Teruhiko Koike, Hiroshi Akima

**Affiliations:** 1 Graduate School of Education and Human Development, Nagoya University, Aichi, Japan; 2 Nippon Sport Science University, Tokyo, Japan; 3 Research Center of Health, Physical Fitness and Sports, Nagoya University, Aichi, Japan; 4 School of International Liberal Studies, Chukyo University, Aichi, Japan; 5 Graduate School of Medicine, Nagoya University, Aichi, Japan; 6 Minami Seikyo Hospital, Aichi, Japan; 7 Present Address: Kyoto Sangyo University, Kyoto, Japan

**Keywords:** Anatomy, Health care, Signs and symptoms

## Abstract

We investigated the effect of physical activity on muscle tissue size and intramuscular adipose tissue (IntraMAT) content in the thigh muscle groups of younger and older men. Twenty younger and 20 older men participated in this study. The muscle tissue cross-sectional area (CSA) and the IntraMAT content in the quadriceps femoris (QF), hamstrings (HM), hip adductors (AD), and mid-thigh total were measured using magnetic resonance imaging. The physical activity time was measured using a triaxial accelerometer, and four levels of physical activity were determined depending on the metabolic equivalent of task (METs), including sedentary (≤ 1.5 METs), light intensity (≤ 2.9 METs), moderate intensity (3.0–5.9 METs), and vigorous intensity (≥ 6.0 METs). No significant correlation was observed between the physical activity parameters and muscle tissue CSA in both groups. The IntraMAT content of the three muscle groups (QF, AD, and HM) and the total thigh was inversely correlated with the time of moderate-intensity physical activity (*r*_*s*_ =  − 0.625 to − 0.489, *P* < 0.05, for all comparisons) in the young group, but not in the older group. These results indicate that IntraMAT accumulation was associated with the amount of moderate-physical activity in younger men.

## Introduction

Age-related decline in muscle strength is known to be caused by muscle atrophy, also called sarcopenia^1–4^. Previous studies reported that a decline in strength is caused by lower muscle quality, such as increased adipose tissue infiltration into the muscle^1^. Two types of adipose tissues that infiltrate the muscle have been defined based on their location: intramuscular adipose tissue (IntraMAT), which is visible extracellular adipose tissue, and intermuscular adipose tissue, which is adipose tissue located between muscle groups^5,6^. The sum of IntraMAT and intermuscular adipose tissue is known as IMAT^6,7^. The IntraMAT content in older individuals has been reported to be twice or more than that of younger individuals in the thigh^8^. Furthermore, excessive IMAT accumulation induces glucose intolerance and insulin resistance^9,10^. An epidemiological study showed that larger muscle cross-sectional area (CSA), lower muscle quality, and higher muscle strength were associated with a lower risk of mortality^11^, suggesting that larger muscle size and lower IntraMAT content in older individuals are desirable to reduce the risk of mortality, insulin resistance and diabetes^3^.

The distribution and amount of adipose tissue are strongly associated with physical activity levels^12,13^. A longitudinal monitoring study demonstrated that inactive twins had 54% higher IMAT in the mid-thigh area than their more active counterparts as a result of different habits after 32 years^14^. Therefore, habitual physical activity is an important factor for the accumulation of adipose tissue in muscles and is independent of genetic factors. These previous findings suggest the importance of maintaining or increasing daily physical activity levels from the viewpoint of preventing IntraMAT accumulation.

Adults are encouraged to maintain moderate-intensity physical activity (MPA: e.g., from 3.0 to 5.9 the metabolic equivalent of task; METs) for 150–300 min per week, vigorous-intensity physical activity (VPA: e.g., ≥ 6.0 METs) for 75–150 min per a week, or a combination of MPA and VPA according to the World Health Organization (WHO)^15^. However, few studies have investigated the relationship between physical activity and muscle mass or quality. A significant correlation was shown between both the number of steps and ≥ 3 METs of physical activity time and lower limb muscle mass in older individuals (aged > 75 years). Interestingly, this correlation was not found for ≤ 3 METs of physical activity time^16^. One study investigated the relationship of physical activity level with IntraMAT. Kent-Braun et al. (2000) reported a significant correlation between the content of IntraMAT in the tibialis anterior and physical activity in older individuals. Using a triaxial accelerometer, daily physical activity levels can be evaluated based on the number of steps, the time of activity with three levels of intensity based on MET, and daily wasted calories. Adipose tissue, specifically lipids, is a highly utilised energy source for light-intensity physical activity (LPA: e.g., ≤ 2.9 METs) compared with VPA^17^. Therefore, LPA time may be inversely correlated with IntraMAT content in young and older individuals.

The purpose of this study was to investigate the relationship of daily physical activity with muscle tissue size and IntraMAT content in the thigh muscle groups of younger and older men. Establishing a relationship between physical activity and muscle tissue size and IntraMAT content is important for understanding the causes of muscle atrophy and IntraMAT accumulation associated with ageing.

## Methods

### Participants

Twenty young (median age 20.5 years, 25th–75th percentile 23.3–27.3 years; younger group) and 20 older (median age 70.0 years, 25th–75th percentile 67.0–72.0 years; older group) men volunteered to participate in the study. Table 1 shows the physical characteristics of the participants. They were widely recruited from healthy and non-obese (body mass index; BMI < 25.0 kg/m^2^) volunteers using a poster in a public facility. This experiment was conducted as a prospective study in 2016 and 2017. Before the experiment, the procedure, purposes, risks, and benefits associated with the study were explained, and written consent was obtained from the participants. The study was approved by the Ethics Committee of the Research Center of Health, Physical Fitness and Sports (28-20) and the Graduate School of Medicine (2016-0254), Nagoya University, and was performed in accordance with the principles of the Declaration of Helsinki.

**Table 1:** Characteristics of the participants.

Characteristic	Younger group	Older group	*P*
Age (years)	20.5 (23.3–27.3)	70.0 (67.0–72.0)	< 0.001
Height (cm)	175.7 (170.9–178.8)	165.5 (161.8–168.5)	< 0.001
Body weight (kg)	63.2 (60.1–73.2)	62.4 (59.3–68.4)	0.529
Body mass index (kg/m^2^**)**	21.3 (19.8–22.6)	23.8 (22.3–24.9)	0.003
Body fat (%)	15.8 (13.3–18.7)	25.5 (24.5–28.7)	< 0.001
**Muscle tissue CSA (cm**^**2**^**)**			
QF	71.6 (63.8–79.9)	54.7 (48.1–59.8)	< 0.001
AD	42.5 (35.0–48.8)	36.6 (31.3–44.8)	0.343
HM	23.9 (20.4–32.1)	16.8 (15.7–19.0)	0.001
Total	131.4 (125.4–162.8)	111.6 (98.9–119.6)	0.001
**IntraMAT content (%)**			
QF	5.9 (3.9–7.6)	9.3 (8.5–12.0)	< 0.001
AD	6.6 (4.6–10.3)	12.5 (10.4–15.9)	< 0.001
HM	13.7 (8.3–15.7)	26.2 (20.4–30.6)	< 0.001
Total	7.5 (5.4–9.6)	13.6 (11.6–15.9)	0.024

Median (25th to 75th percentile), vs. younger group, *CSA* cross-sectional area, *IntraMAT* intramuscular adipose tissue, *QF* quadriceps femoris, *AD* hip adductors, *HM* hamstrings.

### Magnetic resonance imaging (MRI) acquisition

The participants were assessed using a 3.0 T whole-body MRI scanner (MAGNETOM Verio, Siemens Healthcare Diagnostics K.K., Tokyo, Japan). They were placed in the supine position, and images of the thigh were acquired using a body coil. We defined the mid-thigh according to markers attached at the middle point between the greater trochanter and the lateral condyle of the femur. T1-weighted, spin-echo sequence, transaxial images of the right thigh were acquired with the following parameters: three-dimensional, repetition time = 604 ms; echo time = 12 ms; flip angle = 120°; optimised field of view = 256 × 256 mm; slice thickness = 10 mm; and inter slice gap = 0 mm. All participants were instructed to remain as still as possible during imaging.

### Image analysis

Medical Image Processing, Analysis, and Visualization software (version 4.4.0; National Institutes of Health, Bethesda, MD) was used to analyse the images using a personal computer (OptiPlex 7050, Dell Inc., TX, USA). This procedure was essentially the same as that described previously^5,8,18^. First, image heterogeneity caused by suboptimal radiofrequency coil uniformity or gradient-driven eddy currents was corrected using a well-established nonparametric nonuniform intensity normalisation algorithm^8,19^. This step was essential for subsequent analyses that assume a homogenous signal intensity across images. The optimised image correction parameters were determined as follows: end tolerance, 0.0001; maximum iterations, 100; signal threshold, 1; field distance, 25 mm; subsampling factor, 4; kernel full-width half-maximum, 0.15; and wiener filter noise, 0.01. The same parameters were applied to all images.

Next, we calculated the CSA of the IntraMAT and muscle tissue of the thigh using the previously described threshold method with the T1-weighted images^8^. Three regions of interest (ROIs) were isolated in the muscle and subcutaneous adipose tissue areas. Next, an auto-determined threshold was isolated at the base of the first peak of a bimodal histogram. The analysis was repeated three times for each image slice, and the average threshold value was used to classify tissue pixels. The edge of each muscle was carefully traced, and the following parameters were calculated: (1) the total number of pixels within the ROI, (2) the number of pixels with a signal intensity lower than the threshold value (muscle tissue), and (3) the number of pixels with a value higher than the threshold value. Subsequently, the muscle tissue CSA and IntraMAT CSA were calculated using the following equations:

$$\begin{aligned} {\text{Muscle}}\,{\text{tissue}}\,{\text{CSA}}\,({\text{cm}}^{{2}} ) \, & = \, \left( {{\text{muscle}}\,{\text{tissue}}\,{\text{pixel}}\,{\text{number}}} \right) \times \left( {{\text{FOV}}/{\text{matrix}}\,{\text{size}}} \right)^{{2}} \\ {\text{IntraMAT}}\,{\text{CSA}}\,({\text{cm}}^{{2}} ) \, & = \, \left( {{\text{IntraMAT}}\,{\text{pixel}}\,{\text{number}}} \right) \times \left( {{\text{FOV}}/{\text{matrix}}\,{\text{size}}} \right)^{{2}} \\ \end{aligned}$$

in which FOV represents the field of view.

The IntraMAT content was determined using the following equation^8^:

$${\text{IntraMAT}}\,{\text{content }}\left( \% \right) \, = \, \left\{ {{\text{IntraMAT}}\,{\text{CSA/}}\left( {{\text{muscle}}\,{\text{tissue}}\,{\text{CSA }} + {\text{ IntraMAT}}\,{\text{CSA}}} \right)} \right\} \times {1}00$$

For muscle tissue and IntraMAT content, the quadriceps femoris (QF, i.e., the sum of the rectus femoris, vastus lateralis, vastus intermedius, and vastus medialis), hip adductors (AD, i.e., the sum of the adductor longus, adductor brevis, adductor magnus, sartorius, and gracilis), and hamstrings (HM, i.e., the sum of the biceps femoris short head, biceps femoris long head, semitendinosus, and semimembranosus) were evaluated as individual muscle groups. The sum of the CSAs of the QF, AD, and HM was calculated to determine the CSA of the total thigh.

### Physical activity level

Physical activity was measured using an activity monitor equipped with a triaxial accelerometer (Active Style Pro HJA-750C, Omron Healthcare Co., Ltd., Japan). The algorithm for this activity monitor has been described in previous studies^20,21^. The relationship between METs measured by the activity monitor and energy expenditure measured by oxygen uptake and activity (*r* = 0.907, *P* < 0.001) and walking (*r* = 0.961, *P* < 0.001) during housework were examined. It was confirmed that the intensity of physical activity could be estimated with high accuracy^20,21^. All participants were instructed to wear an activity monitor on their left waist throughout the day (except during bathing and sleeping) for 7 consecutive days. The participants were asked to continue their normal daily activities during the measurement period. Non-wearing time was defined as the sum of the time during which the activity count was below the detection threshold and was considered zero for ≥ 20 min^22^. A zero count in the activity monitor indicated that the METs estimated from the average value of the synthetic acceleration was < 0.9. We used data that included measurements from ≥ 600 min per day^22,23^. The intensity of the physical activity times is estimated by the accelerometer and sedentary time is defined as ≤ 1.5 METs, LPA as ≤ 2.9 METs, MPA as 3.0 to 5.9 METs, and VPA as ≥ 6.0 METs^24,25^. The CSV data files from the accelerometer were downloaded using Omron health management software BI-LINK for physical activity professional edition version 2.0 and then processed using custom software (Japan Physical Activity Research Platform Macro program for compiling data). All physical activity parameters were normalised per day.

### Statistical analysis

Prior to data collection, we calculated the minimum sample size required with parameters based on the results of a previous study^13^: correlation ρ (H1), 0.68; significance level (α), 0.05; two-tailed; power (1 − β), 80%, and correlation ρ (H0) = 0. The minimum sample size was estimated as n = 14. For the post-hoc test, the power (1 − β) was 0.601–0.962 in the present study. Our data were not homoscedastic, as determined using Levene’s test. All values are reported as the median and 25th to 75th percentile. The Mann–Whitney U test was used to compare variables between the groups. Spearman’s rank correlation was performed to determine the association between muscle tissue CSA or IntraMAT content and physical activity. The level of significance was set at *P* < 0.05. Statistical analyses were performed using IBM SPSS Statistics software (version 24.0, IBM Japan, Tokyo, Japan) and G* power (version 3.1.9.7).

### Ethics approval

The present study was approved by the Ethics Committee of the Research Center of Health, Physical Fitness and Sports (28-20) and the Graduate School of Medicine (2016-0254), Nagoya University, and was performed in accordance with the Declaration of Helsinki.

### Consent to participate

Before the experiment, the procedures, purpose, risks, and benefits associated with the study were explained and written consent was obtained by the participants.

## Results

Table 1 shows the physical characteristics of the participants. Age, height, and BMI significantly differed between the groups (*P* < 0.001). The CSAs of QF, HM, and total thigh were significantly lower in the older group than in the younger group. Conversely, the CSA of the AD did not significantly differ between the groups. The body fat and IntraMAT content of the thigh muscle groups were significantly higher in the older group than in the younger group.

Table 2 shows the comparison of the physical activity (steps and times) between the younger and older groups. MPA time did not differ significantly and VPA times were lower (*P* < 0.001) in the older group than in the younger group. The step, sedentary, and LPA times did not differ significantly between the groups.

**Table 2:** Comparison of physical activity between the younger and older groups.

Characteristic	Younger group	Older group	*P*
Wear time (min/day)	718.8 (642.0–800.6)	745.8 (682.6–811.0)	0.461
Steps (steps/day)	10,093.3 (6487.0–11,269.6)	8125.4 (5144.3–9963.6)	0.253
**Physical activity time (min/day)**			
Sedentary	565.2 (436.7–632.6)	542.3 (459.0–582.6)	0.495
LPA	802.4 (746.7–863.1)	807.5 (741.7–854.3)	0.989
MPA	27.1 (14.4–45.1)	13.9 (5.1–32.6)	0.052
VPA	3.3 (1.0–6.6)	0.1 (0.0–0.4)	< 0.001
Total	850.0 (770.3–882.5)	827.5 (770.6–886.2)	0.678

Median (25th to 75th percentile), vs. younger group, *LPA* light-intensity physical activity, *MPA* moderate-intensity physical activity, *VPA* vigorous-intensity physical activity.

Table 3 shows the relationships between physical activity and the CSAs of the QF, AD, HM and total thigh. No significant correlations were observed between any physical activity parameter and muscle tissue CSA in either group.

**Table 3:** Correlation between physical activity and muscle tissue cross-sectional area (CSA) in the younger and older groups.

Characteristic	Muscle tissue CSA							
	Younger group				Older group			
	QF	AD	HM	Total	QF	AD	HM	Total
Steps (steps/day)	0.056	− 0.044	− 0.143	0.047	0.221	0.235	0.320	0.260
**Physical activity time (min/day)**								
Sedentary	0.152	0.138	0.406	0.186	0.050	0.286	− 0.137	0.110
LPA	0.141	0.215	0.203	0.202	− 0.087	0.012	− 0.108	− 0.111
MPA	0.047	0.059	0.041	0.126	0.123	0.289	0.085	0.183
VPA	0.397	0.169	0.125	0.269	0.250	0.446*	0.102	0.400
Total	0.159	0.218	0.215	0.229	0.059	0.215	− 0.064	0.075

*P < 0.05, *QF* quadriceps femoris, *AD* hip adductors, *HM* hamstrings, *LPA* light-intensity physical activity, *MPA* moderate-intensity physical activity, *VPA* vigorous-intensity physical activity.

Table 4 shows the relationships between the number of steps, sedentary time, and total physical activity time and IntraMAT content in the three muscle groups and total thigh. In the younger group, but not in the older group, the IntraMAT content of the QF, AD, and total thigh was inversely correlated with the number of steps (*r*_*s*_ =  − 0.709 to − 0.481, *P* < 0.05, for all comparisons). The sedentary time and total physical activity time were not significantly correlated with IntraMAT content in either group.

**Table 4:** Correlation between physical activity and intramuscular adipose tissue (IntraMAT) content in the younger and older groups.

Characteristic	IntraMAT content							
	Younger group				Older group			
	QF	AD	HM	Total	QF	AD	HM	Total
Steps (steps/day)	− 0.709^†^	− 0.481*	− 0.349	− 0.557^†^	− 0.084	− 0.215	− 0.060	− 0.104
**Physical activity time (min/day)**								
Sedentary	− 0.035	0.159	− 0.021	0.078	− 0.197	− 0.170	− 0.197	− 0.206
Total	− 0.427	− 0.153	− 0.113	− 0.211	− 0.268	− 0.156	− 0.286	− 0.286

*P < 0.05, ^†^*P* < 0.01*, QF* quadriceps femoris, *AD* hip adductors, *HM* hamstrings.

**Figure 1:**
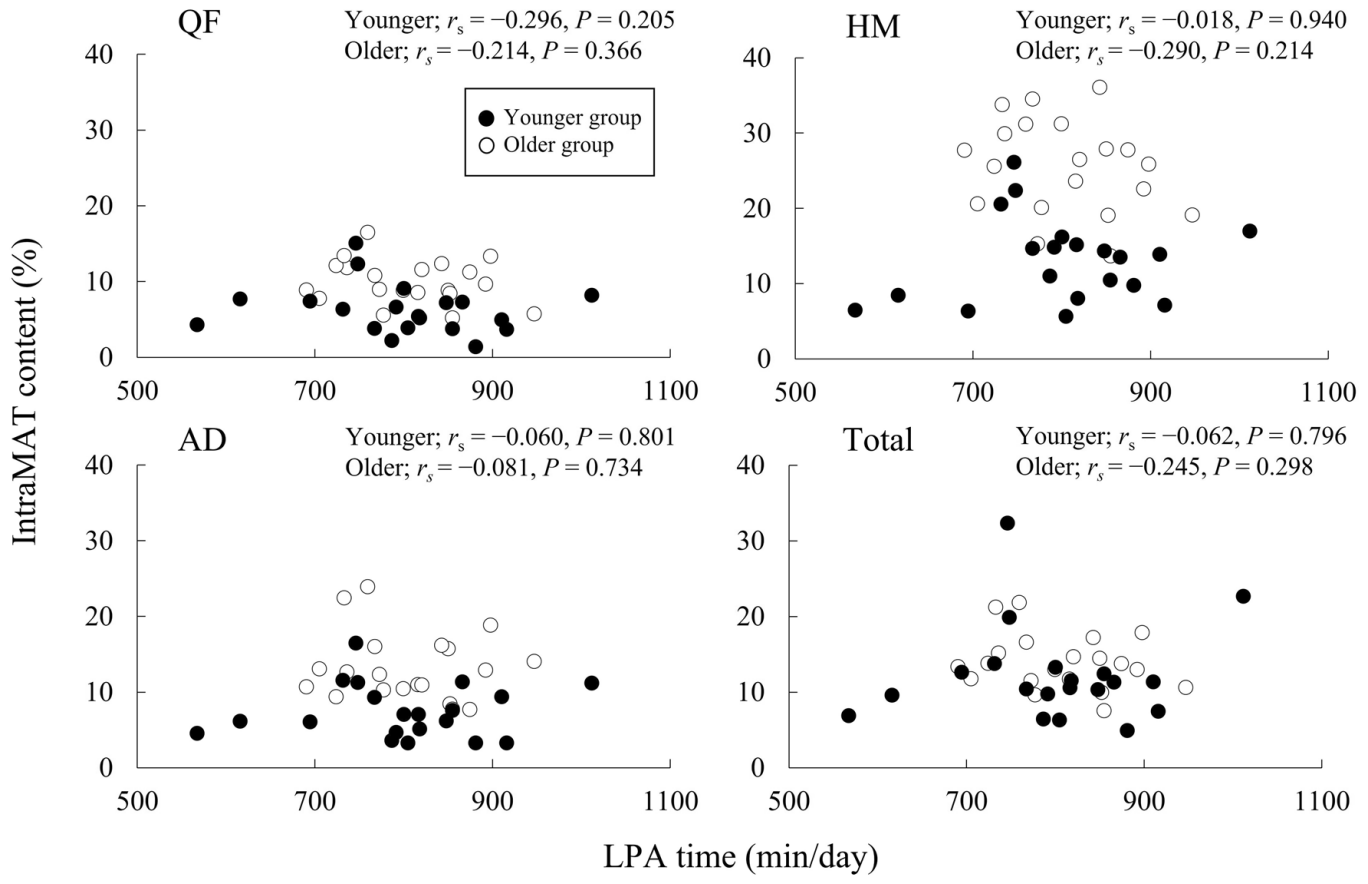
Correlation between light-intensity physical activity (LPA) time and intramuscular adipose tissue (IntraMAT) content in younger and older men. *QF* quadriceps femoris, *AD* hip adductors, and *HM* hamstrings.

Figure 1 depicts that the relationship between the LPA time and the IntraMAT content in the three muscle groups and the total thigh. No significant correlations were recorded between the LPA time and IntraMAT content in either group.

**Figure 2:**
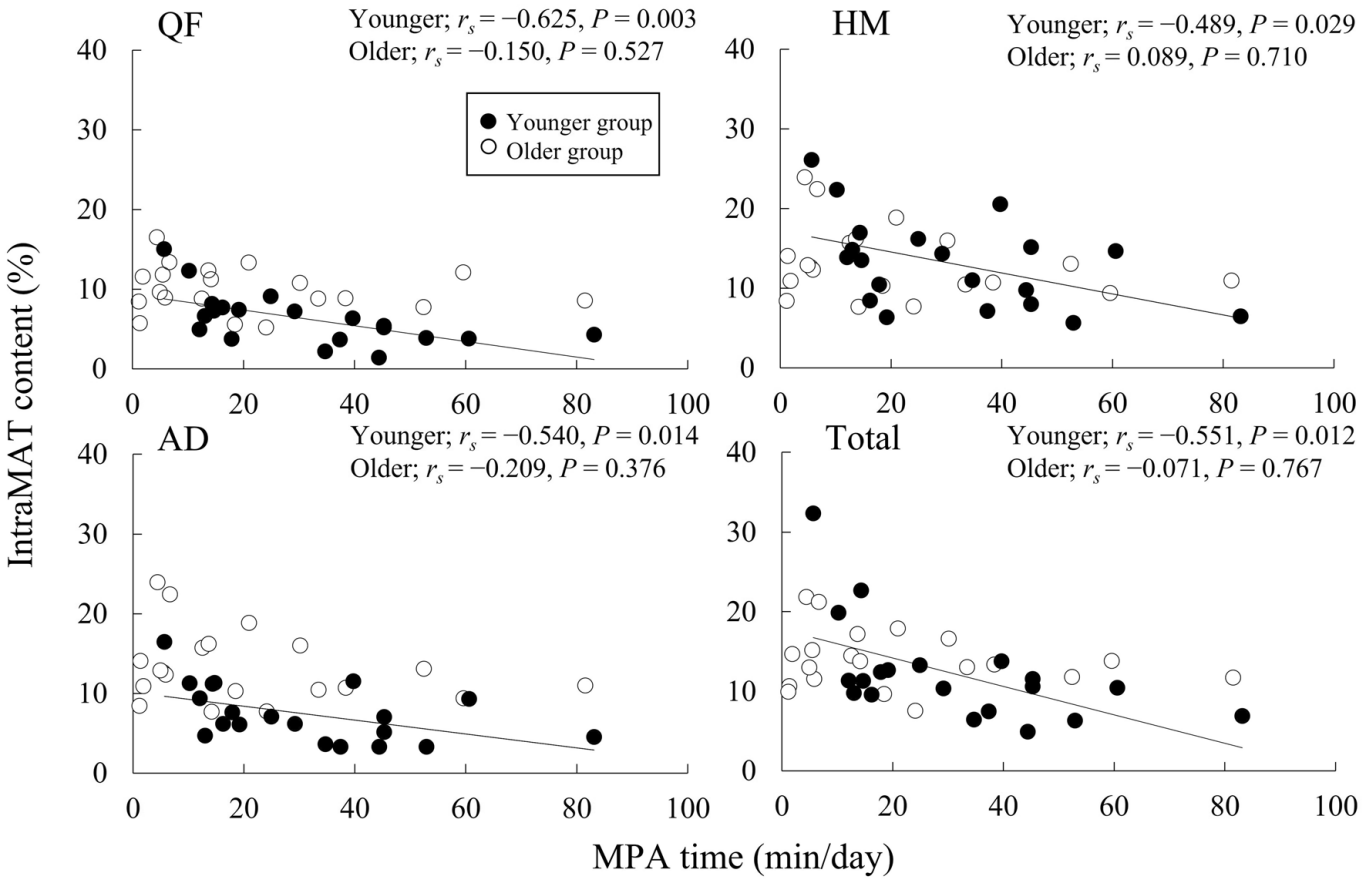
Correlation between moderate-intensity physical activity (MPA) times and intramuscular adipose tissue (IntraMAT) content in younger and older men. *QF* quadriceps femoris, *AD* hip adductors, and *HM* hamstrings.

Figure 2 depicts the relationship between the MPA time and the IntraMAT content in the three muscle groups and the total thigh. In the younger group, the IntraMAT content of the three muscle groups and the total thigh were inversely correlated with the MPA time (*r*_*s*_ =  − 0.625 to − 0.489, *P* < 0.05, for all comparisons). Conversely, no significant correlations were recorded between the MPA time and IntraMAT content in the three muscle groups and the total thigh for the older group.

**Figure 3:**
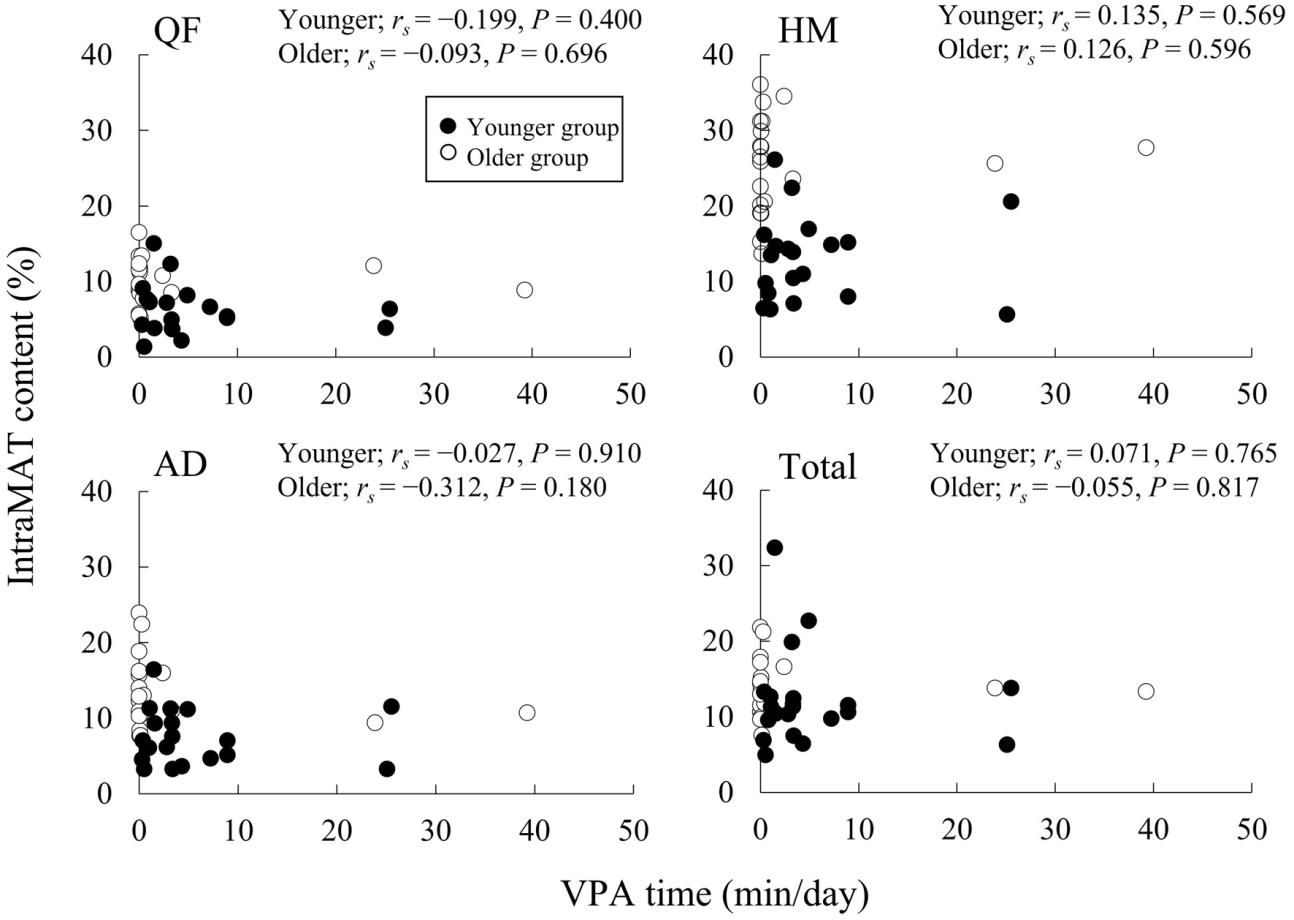
Correlation between vigorous-intensity physical activity (VPA) times and intramuscular adipose tissue (IntraMAT) content in younger and older men. *QF* quadriceps femoris, *AD* hip adductors, and *HM* hamstrings.

Figure 3 illustrates the relationship between the VPA time and the IntraMAT content in the three muscle groups and the total thigh. The VPA time was not significantly correlated with the IntraMAT content in both groups.

## Discussion

We investigated the relationship of daily physical activity evaluated by the number of steps and times with muscle tissue size and IntraMAT content in the thigh muscle groups of younger and older men. The primary findings of this study were: (1) The CSAs of QF, HM, and total thigh were significantly lower in the older group than in the younger group; however, the IntraMAT content of the thigh muscle groups was significantly higher in the older group than in the younger group. (2) The MPA time was inversely correlated with the IntraMAT content of the three muscle groups (QF, AD, and HM) and the total thigh in younger men but not older men.

The WHO guidelines recommend that all adults perform MPA for 150–300 min or VPA for 75–150 min or some combination of MPA and VPA each week to obtain health benefits^15^. Physical activity times in 19 of our 40 participants exceeded the WHO guidelines. In addition, there was no significant difference in the number of steps, sedentary time, and LPA time per day; however, the VPA time was lower in the older than in the younger group (Table 2), although there was no difference in total physical activity time or steps. Moreover, the older group exhibited lower muscle tissue CSA and higher IntraMAT content than the younger group (Table 1), which was consistent with the results of previous studies^26–29^. In to a 5-year longitudinal study, the CSA of the thigh decreased by 4.9% ± 7.4% (1.0% per year), whereas the IMAT CSA increased by 48.5% ± 59.6% (9.7% per year) in older individuals^1^. Therefore, considering the results of this and previous studies, age-related muscle atrophy and an increase in IntraMAT may occur despite similar total physical activity patterns in young and old individuals. In the present study, physical activity (steps and times) showed no significant correlation with muscle tissue CSA in the younger and older groups (Table 3). A previous study reported a negative relationship between the moderate and vigorous physical activity times and lower limb muscle mass in individuals aged > 75 years but not in 64 to 74 aged individuals^16^. The average age of the older group in the present study was 70.7 ± 5.6 years, and only four participants aged > 75 years were included. Considering the results of this and previous studies, physical activity did not affect the muscle mass of the lower limbs of people under 75 years. As resistance training causes muscle hypertrophy even in very old individuals^30^, this indicates that the suppression of muscle atrophy with age requires resistance exercise even in older individuals who have similar activity levels to those of younger individuals.

There were noteworthy findings regarding the relationship between physical activity and IntraMAT content in the present study. The IntraMAT content in the total thigh and in each muscle group was inversely correlated with steps and MPA time but not with sedentary, LPA, and VPA times in the younger group, and surprisingly no significant relationship was seen between physical activity and IntraMAT content in the older group (Figs. 1, 2, and 3). These results were not consistent with the results of previous studies^12,13^. For example, Kent-Braun et al. (2000), in contrast to our observations, reported a negative correlation between IntraMAT content in the tibialis anterior and physical activity in older individuals but not in younger participants. The explanation for these conflicting results is unclear. However, there are major differences in the sex and measurement sites between Kent-Braun et al. (2000) and this study. Kent-Braun et al. (2000) investigated the tibialis anterior of the lower leg in both sexes (11 men and 10 women), but this study investigated the thigh muscle groups in only men. IntraMAT content depends on the individual muscle^8,31^; therefore, the differences between the measurement sites may explain the differences from Kent-Braun et al. (2000).

Akima et al. (2015) reported that IntraMAT content in the total thigh was inversely associated with muscle tissue CSA normalised by body weight in younger and older individuals^8^. In line with this observation, we observed that the muscle tissue CSA was inversely correlated with IntraMAT content in the QF and HM (*r*_*s*_ =  − 0.591 and − 0.530, *P* = 0.006 and 0.016), but in older individuals only. Considering the results of this and previous studies^8^, we speculate that age-related changes in energy substrate utilisation, in addition to age-related muscle atrophy, contribute to IntraMAT accumulation. Lipids are utilised at a higher rate during low-to-moderate-intensity physical activity compared with high-intensity physical activity^17^. In addition to intensity, the contribution of carbohydrates and fats as fuel during exercise is dependent upon duration of exercise as well as age^32^, sex^33^, training status^34^, and nutrition^4^. Sial et al. (1996) showed that fat oxidation during exercise was lower in older individuals compared with young individuals who exercised at either the same absolute or similar relative intensities^35^. Such age-related changes in fat oxidation during exercise might affect IntraMAT accumulation in older individuals.

There are several limitations to the present study. First, the statistical power (1 − β, α = 0.05) was medium-to-high (0.601–0.962); larger studies are needed to further address the effects of age, sex, disease, and BMI on our findings. Only healthy and non-overweight or obese men participated in the study. Second, our study had a cross-sectional design; therefore, we could not affirm whether the lack of MPA resulted in IntraMAT accumulation in younger men. Further longitudinal research is needed to investigate the effect of MPA on IntraMAT content.

## Conclusion

Significant negative correlations were observed between the IntraMAT content of the thigh muscle groups and triaxial accelerometer-determined physical activity levels, especially the duration of moderate-physical activity in younger men, but not in older men. These results suggest that IntraMAT accumulation may be associated with the amount of moderate-physical activity in younger men. Furthermore, the reasons for the difference in the results between the younger and older men was difficult to clarify in this study. Further longitudinal research is needed to investigate the effects of ageing and physical activity on IntraMAT content.

## Data Availability

The data that support the findings of this study are available on request from the corresponding author (M.O.). The data are not publicly available because they contain information that could compromise the privacy of the research participants.

## Abbreviations

AD: Hip adductors
BMI: Body mass index
CSA: Cross-sectional area
HM: Hamstrings
ROI: Region of interest
LPA: Light-intensity physical activity
MET: Metabolic equivalent of task
MRI: Magnetic resonance imaging
MPA: Moderate-intensity physical activity
IMAT: Intramuscular and intermuscular adipose tissues
IntraMAT: Intramuscular adipose tissue
QF: Quadriceps femoris
VPA: Vigorous-intensity physical activity
